# Traditional knowledge on herbal drinks among indigenous communities in Azad Jammu and Kashmir, Pakistan

**DOI:** 10.1186/s13002-018-0217-8

**Published:** 2018-02-21

**Authors:** Neelam Rashid, Rodrigue Castro Gbedomon, Mushtaq Ahmad, Valère Kolawolé Salako, Muhammad Zafar, Khafsa Malik

**Affiliations:** 1 Department of Botany, Mirpur University of Science and Technology, Mirpur, Azad Kashmir Pakistan; 2 Laboratoire de Biomathématiques et d’Estimations Forestières, Faculty of Agronomic Sciences, University of Abomey-Calavi, 04, BP 1525 Cotonou, Benin; 3 Department of Plant Sciences, Quaid- I-Azam University, Islamabad, Pakistan

## Abstract

**Background:**

Traditional knowledge about the use of medicinal plants for herbal drinks (HDs) is not well documented in the Azad Kashmir region despite their widespread use. This study highlights the taxonomic diversity and traditional knowledge on medicinal plants used for HDs while examining the diversity of diseases treated with HDs in the study area.

**Methods:**

Individual discussions were conducted with 255 informants (84 women and 171 men). Data gathered included (i) informant age and gender, (ii) HD species and respective plant parts used, (iii) health disorders treated, and (iv) mode of preparation and utilizations. Quantitative ethnobotanical indices including relative frequency of citation (RFC), informant consensus factor (ICF), and use value (UV) were used for data analyses.

**Results:**

Altogether, 73 medicinal plants belonging to 40 families and 66 genera were reported to be used in HD preparations, with Asteraceae being the richest family. The average number of HD species cited was 9.09 ± 0.17 per informant and did not vary either by age or gender. In addition, men and women, and adults and the young used the same pool of species (dissimilarity nearly zero). The most used plant parts were leaves (20.00%), roots (17.25%), and fruits (16.47%). Based on UV, the top five most used species were *Valeriana jatamansi*, *Isodon rugosus*, *Onopordum acanthium*, *Acacia nilotica*, and *Viola canescens*; and the UV was similar among gender and age categories too. The most utilized herbal preparation forms included decoctions, infusions, and tea. One hundred and eleven diseases grouped into 13 ailment categories were reported to be cured using HDs. The main category of disease treated with HDs was gastrointestinal (GIT) disorders (RFC = 17.43%). Relatively few species were used by a large proportion of informants for each category of ailment (ICF ≥ 0.60). Only one species was used for “glandular disorders” and “eye diseases” (ICF = 1).

A novelty of about 22% (16 species) was recorded for HD species in the present work.

**Conclusion:**

The diversity of medicinal plant species used as HDs and the associated traditional knowledge are of considerable value to the indigenous communities of the Azad Kashmir region. Therefore, there is a need for conservation and preservation of medicinal HD species as well as the wealth of indigenous knowledge. The conservation effort should be high for species in the ailments categories glandular disorders and eye diseases. The therapeutic uses of HDs have provided basic data for further research focused on phytochemical and pharmacological studies and conservation of the most important species.

## Background

Traditional medicine (TM), also known as complementary and alternative medicine in developed countries, is widely used and is of rapidly growing interest in health care systems all over the world. About 80% of the world’s population, particularly in developing regions, relies on TM practices to meet their health care needs [1, 2]. TM is very popular and attracts much attention from a large spectrum of health system stakeholders, not only for its accessibility and affordability for poor people but also because of the risk of adverse effects of chemical drugs in allopathic medicine. As a recognition of its importance and as a response to skepticism and disbelief from some stakeholders, in 2002, the World Health Organization defined a strategy to address issues of policy, safety, efficacy, quality, access, and rational use of TM [3]. TM includes medication therapies and non-medication therapies. Whereas the latter are carried out primarily without the use of medication, the first involve the use of herbal medicines [4–6], animal parts [7–10], and minerals [11, 12].

Among the medication therapies, herbal medicine or phytotherapy is encountered worldwide, and its use is very ancient. Human use of plants as medicines was dated to at least the Middle Paleolithic age some 60,000 years ago [13, 14]. The Himalayan region comprised of Afghanistan, Bangladesh, Bhutan, China, Myanmar, Nepal, Pakistan, and India is reputed to be a rich storehouse and hotspot of valuable medicine plant species. About 1748 plant species corresponding to 21.85% of the local 8000 angiosperm species are used for medicinal purposes [15].

In Pakistan, the flora is quite rich and is estimated to consist of about 6000 species of higher plants of which 600 to 700 species, the majority growing in the wild, are used for medicinal purposes [16]. Throughout the country, TM using plant species is popularly practiced among a large segment of its population [17–20]. Eighty percent of people belonging to the rural areas still depend upon the herbal medicines in Pakistan. The most realistic and commonly employed therapy for diseases is to make infusions or decoctions from different plant parts [21]. Local market systems named “Pansara” specifically dealing with medicinal business have been reported [16, 22–24], with important quantities of plants exchanged locally or exported. For some years, important concerns due to excessive use of herbal medicine have arisen and are related to the conservation issue of medicinal plant species [16] and adulteration of botanical medicine [24]. Indeed, the majority of medicinal plants used for the herbal drug industry and local communities come from wild collection. Overexploitation and unsuitable collection methods are contributing to the extinction of some medicinal plants and bringing others to the brink of extinction [16]. Beyond the conservation issue, the excessive use of medicinal plants may lead to the misidentification of adulterant plants, hence compromising the quality control and standardization [25]. Moreover, ethnobotanical medicine in Pakistan is still a huge field of investigation. The last review on medicinal plants in Pakistan [25] evidenced the gaps of knowledge on herbal medicine and called upon extensive research. This paper aims at contributing to this ongoing body of knowledge in herbal medicinal, focusing on HDs.

HDs refer to beverages made from the infusion or decoction of herbals, spices, fruits, or other plant materials, served cold or hot. They include herbal teas, fruit drinks, infusions, and decoctions. HDs are highly appreciated mostly because of their therapeutic purposes [26]. In Pakistan, the available literature on medicinal plants used for HDs is recent and preliminary [2, 27], and important issues remain unanswered and insufficiently documented. Using the region of Azad Jammu and Kashmir in Pakistan, this study aims at:

1. Assessing the taxonomic diversity of plants used for HDs. The medicinal plant richness of Pakistan is estimated to be about 600 to 700 plant species [25]. What is the taxonomic composition of the pool selected for HD preparation? Based on the non-random selection theory [28, 29], which predicts that medicinal plant selection is not random, it is expected that the number of medicinal plant species used for HDs differs across botanical families.
2. Assessing the traditional knowledge on HDs and its relationship with age and gender. Indeed, the use of medicinal plants is based on trial and error and is passed on from one generation to another, after refinement and additions [30]. What is the traditional knowledge (species uses, plant parts used, mode of preparation, etc.) related to HD preparation? Because various individual socio-cultural and demographic traits, mainly gender and age, are correlated with an individual’s level of plant knowledge [31–33], we expected traditional knowledge related to HDs to be associated with age and gender. With women and older people tending to have a greater knowledge of local medicinal flora [31, 34], we hypothesized that traditional knowledge on HDs is mostly held by women and old people in Pakistan.
3. Assessing the diversity of diseases treated by HDs. About 10–12% of plants in Pakistan are used to meet health care needs. In the special case of HDs made of medicinal plants, what diseases are treated, and which plants are solicited? We expected some diseases to be treated by a restricted set of plants.

## Methods

### Study area

**Fig. 1:**
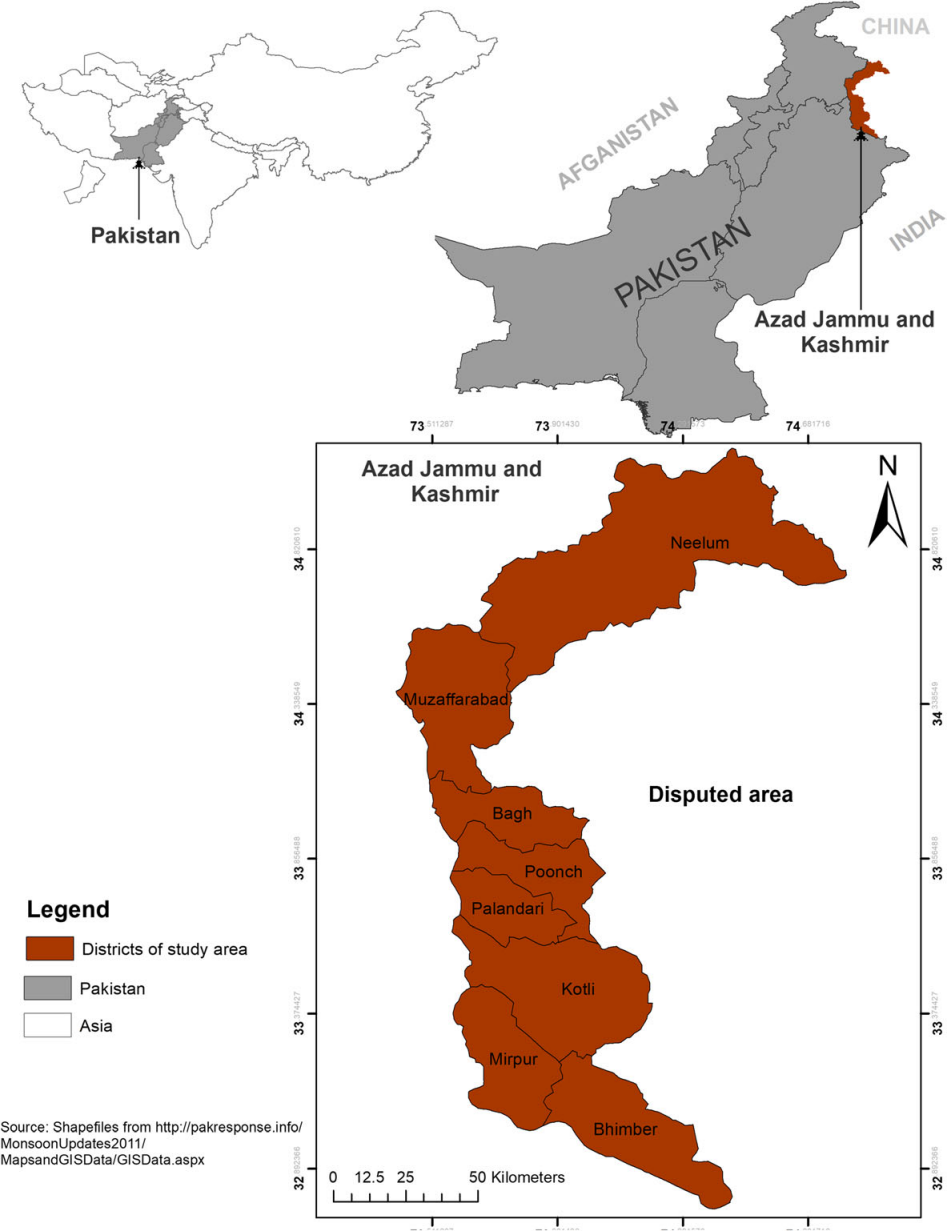
Map of Azad Jammu and Kashmir

The region of Azad Kashmir is positioned between 34°22′ North latitude and 73°28′00 East longitude. The total area is about 13,297 km^2^ [35]. The study sites included eight districts; Muzaffarabad, Mirpur, Kotli, Bagh, Poonch, Palandari, Neelum, and Bhimber. The region of Azad Jammu and Kashmir is characterized by a craggy landscape, mountains, waterfalls, paddocks, river, streams, plains, and forests [36] (Fig. 1).

The region of Azad Jammu and Kashmir has varied climatic characteristics with temperate, humid, sub-tropical, and sub-alpine eco-regions. Due to peculiar geographical and climatic conditions, this area is gifted with a rich floral diversity [37]. The local inhabitants of Azad Jammu and Kashmir belong to various cultures and speak different local or native languages such as Pahari, Pothohari, Hindko, and Gojri, and the Urdu language is also well-known to the local people.

### Ethnographic and socio-economic characteristics of the Azad Jammu and Kashmir region

Overall in the Azad Jammu and Kashmir region, Mughals, Syed, Raja, Chaudhary, and Maliks are the prominent castes. Azad Kashmir is generally considered as a Pahari speaking region. There is a large Gojri community and some pockets of Kashmiri, especially in Neelam Valley regions. Hindu and Punjabi are also spoken in Muzaffarabad and Bhimber areas, respectively, adding to the cultural diversity of this region. Urdu enjoys the status of official language in the region. Kashur, the language of Kashmir, is spoken by families residing in different parts of Azad Kashmir [38]. The population of the valley is approximately over 4 million. The population density in Azad Kashmir is estimated at 343.5 inhabitant/km^2^.

Internally, the region of Azad Jammu and Kashmir is organized into three administrative divisions including Mirpur, Poonch, and Muzaffarabad. The divisions are further divided into 10 districts and 30 sub-districts or Tehsils. Most of the Kashmiris live in villages and are dependent on agriculture. Local people are primarily affiliated with agriculture and also rear livestock. The plain areas of this region have an agricultural economy primarily dependent on rainfall. Maize, wheat, and rice are the main crops, and beans and peas are also cultivated in the area. Tourism has greatly improved the socio-economic conditions of the area by providing job opportunities to local people. Local people work in hotels and restaurants, as guides and jeep drivers, and some have opened shops at tourist resorts.

### Sampling and data collection

#### Sampling

The sampling method combined both probabilistic and non-probabilistic approaches. First, 5 villages were purposely chosen in each one of the 8 districts of the study area, for a total of 40 villages. The main criterion of selection was the prevalence of herbal medicine practice. Second, for each district, a sub-sample size of about 30 to 35 informants was chosen, for a total of 255 informants for the whole study area. A sample size of a minimum of 30 informants per district is the minimum size for a good approximation of the normal distribution [39] and for sound statistical analyses. Before entering each of the study areas, permission was sought from a local area leader after explaining the objectives of the study. From this leader, we took the name of the first key informant, whereas the rest of the respondents were selected by a snowball sampling technique [40]. Out of the 255 interviewed persons, 76 % were male, and the remaining 24% were female. Thirty percent of informants were under 40 years old.

#### Data collection

The collection of data was undertaken by an experienced researcher who had a strong knowledge of the local languages and culture of Azad Jammu and Kashmir. Ethnobotanical data were collected during individual interviews and group discussions with informants. Questions asked during the interviews were related to socio-demographic characteristics of informants (name, age, gender, occupation, area, etc.) and plant information (local name of species, habitat, part used, disease treated, mode of preparation and utilization, dose of application, duration of treatment, status of the species, etc.). Interviews were conducted in the local language and lasted on average 15 to 30 min depending on interview conditions.

The specimens of the plants used in HDs were collected and preserved using herbarium techniques as suggested by [41]. Vouchers were first identified using the Flora of Pakistan and were sent to the National Herbarium of Pakistan (http://herbarium.qau.edu.pk/) in Islamabad for formal identification and authentication.

### Concept clarification

Herbal medicine, also known as herbalism or botanical medicine, is a medical system based on the use of plants or plant extracts to treat illness and to assist bodily functions. Sometimes, herbal remedies are prepared as drinks by following different techniques and are served hot or cold. They include herbal teas, fruit drinks, infusions, and decoctions prepared for medicinal purposes. This definition excludes “recreational tea” [26] and other beverages prepared as an infusion or decoction that are consumed primarily in a food fortification context or for their general socio-cultural and recreational value. There are different modes of preparation/utilizations of HDs (Table 1).

**Table 1:** Definition of some concepts related to herbal drinks

Herbal drinks	Definition
Herbal tea (HT)	An herbal beverage made with flowers, leaves, and soft stems of plants. Herbal tea is light combining both recreational and medicinal purposes.
Herbal infusion (HI)	An herbal tea made from longer steeping or boiling with a larger amount of herb. Herbal infusion is exclusively used for medicinal purpose and to provide body with high dose of vitamins and minerals. Infusion is intended to extract vitamins and volatile ingredients from the leaves and flowers.
Herbal decoction (HD)	An herbal beverage made with roots, barks, seeds, rhizomes, and woods and intended to extract mineral salts and bitter principles from plants. Unlike infusions, decoctions are left on the heat and simmered for a length of time.
Herbal fruit juice (HFJ)	An herbal beverage made with fruits.

### Ethical consideration

The data were collected with critical care by keeping in view the cultural values of local communities. Respondents were informed that the study was carried out for academic reasons and not for commercial purposes. Finally, informants acknowledged the concept and reached an agreement. Prior, informed consent was obtained for conducting interviews, and the researchers involved in the conceptualization of this study adhered to the ethical guidelines of the International Society of Ethnobiology [42].

### Statistical analyses

Quantitative ethnobotanical indices including relative frequency of citation (RFC), informant consensus factor (ICF), and use value (UV) were used for data analyses.

#### Taxonomic diversity of plants used in HDs by communities in Azad Jammu and Kashmir

The taxonomic plant diversity was assessed through the calculation of the species richness, genera richness, and family richness. The RFC of species, expressed as the ratio (in %) between the numbers of informants who cited the species divided by the total number of surveyed informants, was used to identify the most frequently cited species.

#### Local traditional knowledge on HDs in Azad Jammu and Kashmir

##### Gender and age differences in the knowledge of HD species

The species used for HDs were compared between men and women and between informants aged less than 40 years and informants aged 40 years and older using both quantitative and qualitative aspects. The rationale for this analysis was that number of species used may be similar, whereas the species used could be quite different. A Poisson generalized linear model (GLM) was used to assess the relationship between the number of species cited on one hand, and gender and age category and their interaction on the other. Analysis of similarities (ANOSIM) [43] was used to test whether there was a significant difference between men and women or informants aged less than 40 years and those aged 40 years and older with respect to species used. ANOSIM provides a way to test whether there is a significant difference between two or more groups of sampling units. ANOSIM operates directly on a dissimilarity matrix and is philosophically allied with non-metric multidimensional scaling ordination [44] in that it uses only the rank order of dissimilarity values. If two groups of sampling units are really different in their species composition, then compositional dissimilarities between the groups ought to be greater than those within the groups. The ANOSIM statistic *R* is based on the difference of mean ranks between groups. It ranges from 0 to 1, 0 indicating a completely random grouping and 1 indicating a completely different species composition.

##### Plant parts used for HDs

First, a list of plant parts used for HDs was established. Next, the RFC of each plant part expressed as the proportion of informants who mentioned that plant part was computed as a measure of informant consensus on its use in an HD.

##### Preparation and mode of utilization

The diversity of the preparation mode of HDs and its occurrence among informants was calculated using RFC. The utilization mode reported by informants was then described.

#### Medicinal uses of HDs in Azad Jammu and Kashmir

##### Informant consensus on medicinal uses of HDs

To determine informant consensus on the spectrum of species used for each category of ailment, the ICF was calculated. Here, ICF estimates the relationship between the “number of use-reports in each category (*n*_ur_) minus the number of species used (*n*_t_)” and the “number of use-reports in each category minus 1,” as described in Eq. 1.

1 $$ \mathrm{ICF}=\frac{n_{\mathrm{ur}}-{n}_{\mathrm{t}}}{n_{\mathrm{ur}}-1} $$

Values of ICF range from 0 to 1. A high value (close to 1) indicates that relatively few taxa (usually species) are used by a large proportion of people, whereas a low value indicates that the informants disagree on the taxa to be used in the treatment within a category of illness.

##### UV of HD plant species and relationship with gender and age category of informants

The importance of each single species in HDs was estimated using the relative UV [45], which is a modified version of the UV method introduced by Phillips and Gentry [46]. This modified version of UV (Eq. 2) captures all the known uses by an individual:

2 $$ \mathrm{UV}=\sum \limits_{i=1}^n\frac{\mathrm{U}{\mathrm{R}}_{\mathrm{i}}}{n} $$

UR_i_ is the number of use-reports mentioned by informant i. n is the number of informants.

UV was used to identify the top 14 species in HDs, i.e., species with the highest values of UV. The data showed that for all top 14 species, each informant mentioned either one or no use of each single species (i.e., no informant mentioned more than one use for each single species). Therefore, a binomial logistic model was used to assess whether the UV of each of the top 14 species was related to gender and age category. The full model was first specified, and then a stepwise selection method based on AIC (Akaike information criterion) was used to select the parsimonious model.

All statistical analyses were implemented in R software [47], and the significance level was considered at alpha = 0.05.

## Results

### Taxonomic diversity and life form of plants used in HDs in Azad Jammu and Kashmir

**Fig. 2:**
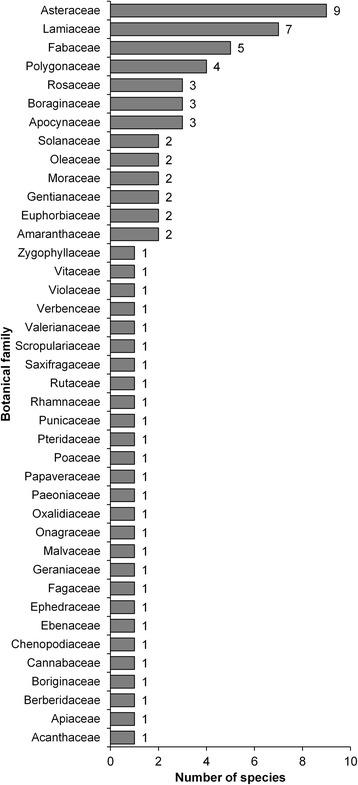
Family rank of species used in herbal drinks in Azad Jammu and Kashmir

Seventy-three species belonging to 40 families and 39 genera were cited as being used for HDs. The richest family was Asteraceae (9 species) followed by Lamiaceae (7 species) and Fabaceae (5 species). Twenty-eight families had only one species, whereas seven families had only two species (Fig. 2). The most common genera reported were *Solanum*, *Morus*, and *Mentha*.

The 73 reported plant species were dominantly herbs (69.86%). The remaining were comprised of shrubs (16.43%), trees (12.35%), and climbers (1.36%).

### Local traditional knowledge on HDs in Azad Jammu and Kashmir

#### Gender and age differences in the knowledge of HD species

**Fig. 3:**
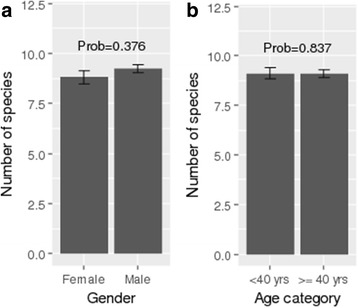
**a** Diversity of plant species used in herbal drinks across gender. **b** Diversity of plant species used in herbal drinks across age categories

Quantitatively, the number of cited species per informant varied from 1 to a maximum of 17. The number of reported species did not differ either between gender (df = 1, deviance = 0.78, prob. = 0.376) or between age categories (df = 1, deviance = 0.042, prob. = 0.837). Although not significant, men cited on average more species (9.23 ± 0.21) than women (8.82 ± 0.32) (Fig. 3a). The average number of cited species was 9.08 ± 0.21 for informants aged less than 40 years and 9.12 ± 0.29 for informants aged 40 years and older (Fig. 3b).

**Fig. 4:**
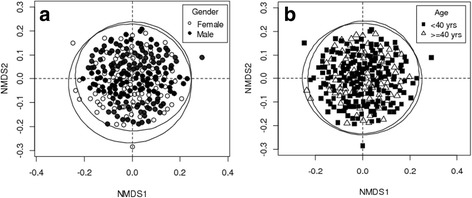
**a** Similarity of plant species used in herbal drinks across gender. **b** Similarity of plant species used in herbal drinks across age categories

From a qualitative perspective, the ANOSIM R statistic was − 0.0045 (prob. = 0.613) and 0.0083 (prob. = 0.225), respectively, for age and gender, indicating that both men and women or adults and the young used the same pool of species for HDs as illustrated by the quasi overlapping of the confidence ellipses (see Fig. 4).

#### Plant parts used in HD preparation

Overall, several parts of the plant were used in HDs, including leaves, roots, stems, fruit, seeds, rhizomes, and bark. Leaves was the most cited plant part (20.00%) followed by roots (17.25%) and fruits (16.47%). Seeds and rhizomes were the least cited plant parts and grouped in the category “others.”

#### Preparation and modes of utilization of HDs

Generally, HDs are prepared using simple methods depending on the plant material. The utilization modes included tea, juice, infusion, decoction, and beverage. The main form of utilization reported by informants was decoction. Decoctions are easier to make because plant parts are only to be boiled with water. Mostly, fresh plant parts are preferably used in preparation of HDs as reported by informants, and dried parts are also used. Informants reported that drying the plant parts may reduce the effectiveness of bio agents, because the ingredients present are damaged to some extent. Almost all preparations reported in the study (Table 2) were made from a single plant species with addition of sugar, water, and in some cases honey to increase the taste.

**Table 2:** Ethnomedicinal uses of herbal drinks in Azad Kashmir

Scientific name	Voucher no in Pakistan NH	Local name	Life form	Plant part used	Administration	Disease treated††	Preparation mode	Active constituents [literature reference]	UV	RFC
*Acacia nilotica* (L.) Willd. ex Delile Fabaceae	ISL −49,177	Keekar	Tree	Bark	Decoction	**Toothache**	Bark is ground to powder and put into boiling water. Decoction prepared is used for gargling in case of toothache.	Alkaloids, saponins, anthraquinones [102]	0.03	0.09
*Achillea millefolium* L. Asteraceae	ISL-40550	Rattiboti	Herb	Leaves, flower	Decoction, infusion, tea	Brain disorders, fever, **piles**, headache, stomach disorders	Boiling water is poured over dried leaves, and this hot infusion is taken to induce sweating that relieves fever. Tea is made by dissolving plant material in boiled water and sugar can also be added. Tea is consumed in case of stomach disorders.	Tannins, flavonoids, saponins [103]	0.16	0.27
†*Adiantum venustum* D. Don Pteridaceae	ISL-100609	Kakwa	Herb	Leaves,roots	Infusion	**Fever**	Infusion of leaves is prepared by pouring hot water over leaves of plant. Sugar can also be added. This infusion is taken twice a day to relieve fever.	Flavonoids, terpenoids, steroids [104]	0.08	0.14
*Aerva javanica* (Burm.f.) Juss. ex Schult. Amaranthaceae	ISL-50064	Boikalan	Herb	Leaves	Infusion	**Stomach disorders**	Leaves are infused in hot water which is then consumed to get ease from stomach disorders.	Terpenoids, alkaloids [105]	0.01	0.12
*Ajuga integrifolia* Buch.-Ham. ex D.Don Lamiaceae	ISL-109327	Kauri booti	Herb	Leaves, stem	Decoction	**Jaundice**	1–2 g of dried leaves is boiled in water. The decoction prepared is taken in early morning before breakfast to treat jaundice.	Glycosides, terpenoids [106]	0.01	0.09
*Amaranthus viridis* L. Amaranthaceae	ISL-115671	Ganhar	Herb	Leaves	Decoction	**Anemia**	Leaves of plant are boiled in water for 1 h to make decoction at slow heat. Half cup of decoction is then taken once a day in case of anemia.	Tannins, flavonoids, glycosides [107]	0.03	0.13
*Arnebia benthamii* (Wall. ex G.Don) I.M.Johnst. Boraginaceae	ISL-94123	Ghaozban	Herb	Root	Tea	Pneumonia, **flu**	Tea is prepared from roots of plant and taken three times a day in case of pneumonia.	Phenolics, steroids [108]	0.05	0.09
*Bauhinia variegate* L. Fabaceae	ISL- 54078	Kachnar	Tree	Leaves, flowers	Infusion	**Inflammation**	Leaves and crushed flowers are soaked in water for 2 days then filtered. Hot water is poured over this filtrate which is used against inflammation.	Alkaloids, saponins, phenolics, tannins, saponins [109]	0.03	0.15
*Berberis lycium* Royle. Berberidaceae	ISL-108676	Sumbal	Shrub	Fruit, root	Infusion, juice, decoction	Cough, **jaundice**, Sore throat	Grinded root bark mixed with water, and sugar is taken before sleeping in sore throat and pain Juice of fruit is used in gum troubles.	Phenolic, flavonoids [110]	0.05	0.11
*Bergenia ciliata* (Haw.) Sternb. Saxifragaceae	ISL 100171	Batwaya	Herb	Rhizome	Infusion	Earache, **muscular pain**	Rhizome is grinded and 1–2 teaspoon of powder is infused in boiling water which is taken before breakfast for muscular pains.	Alkaloids, flavovoids, phenolics, glyscosides saponins [111]	0.1	0.14
*Cannabis sativa* L. Cannabaceae	ISL-101521	Bhang	Herb	Leaves	Beverage	**Mental relief**	Leaves and flowering tops are mixed with milk with addition of sugars and almonds to make drink sardai which helps in mental relaxation.	Terpenes, flavonoids [112]	0.02	0.08
*Carissa spinarum* L. Apocynaceae	ISL-22557	Granda	Shrub	Leaves	Infusion	**Jaundice**	Infusion of leaves is taken orally in early morning to treat jaundice.	Flavonoids [113]	0.04	0.05
*Chrysojasminum humile* (L.) Banfi Oleaceae	ISL-116143	Chambeli	Shrub	Root	Decoction	**Ringworm**	Roots are boiled in water. Decoction obtained is cooled for 10–15 min and then consumed orally in case of ringworm.	Glucoside, phenolics [114]	0.07	0.2
*Cichorium intybus*l. Asteraceae	ISL-116352	Handh	Herb	Flowers, leaves, root	Decoction	**Bronchial disorders**	2–3 g of flowers and leave’s material is boiled in water and given orally at least once in a day to get ease in respiratory disorders.	Alkaloids, flavonoids, saponins [115]	0.02	0.08
*Corydalis incisa* (Thunb.) Pers. Papaveraceae	ISL-116140	Papra	Herb	Whole plant	Decoction, juice	Fever, **blood purifier**	Ground plant material is taken, and water is added in it. The juice is then extracted which is used to relieve fever.	Tannins, alkaloids [116]	0.06	0.07
*Cymbopogon citratus* (DC.) Stapf Poaceae	ISL 48939	Serai	Shrub	Leaves	Tea	**Stomach disorders**, headache	Leaves are infused in hot water for 20 min to make tea which is given to treat stomach pain.	Alkaloids, saponins, tannins [117]	0.13	0.09
*Cynoglossum lanceolatum* Forssk. Boraginaceae	ISL-196130	Lainda	Herb	Root	Decoction	**Joint pain**	Decoction of root is taken for rheumatic disorders.	Alkaloids, flavonoids, triterpenes [118]	0.04	0.16
*Diospyros lotus* L. Ebenaceae	ISL-68699	Kala Amlook	Tree	Fruit	Juice	Stomach disorders	Juice of fruit is extracted and consumed in case of stomach problems.	Tannins, alkaloids [119]	0.05	0.35
*Ephedra gerardiana* Wall. ex Stapf Ephedraceae	ISL-67687	Asmanipoda	Herb	Root	Decoction	**Joint pain**	Roots decoction is taken twice a day to treat rheumatism.	Alkaloids, tannins, glycosides [120]	0.03	0.23
†*Euphorbia illirica* Lam. Euphorbiaceae	ISL-68688	Kankoli	Shrub	Leaves	Infusion	**Urinary disorders**	Leaves are infused in hot water and taken orally to help treat urinary problems.	Alkaloids, flavonoids [121]	0.01	0.07
*Euphorbia prostrata* Aiton Euphorbiaceae	ISL-65674	Hazardaani	Herb	Stem	Decoction	**Dysentery**, diarrhea	Stem is chopped into pieces and boiled in water then cooled for 1 h. Decoction is given orally after intervals to treat dehydration problems.	Tannins, glycosides [122]	0.08	0.13
†*Fagonia cretica* L. Zygophyllaceae	ISL 47236	Temaan	Shrub	Leaves	Infusion	**Cough**	Infusion of leaves is taken to get relief from cough with regular intervals of hours.	Terpenoids, saponins [123]	0.02	0.07
† *Fagopyrum esculentum* Moench Polygonaceae	ISL-116233	Hulla	Herb	Root	Juice	**Fever**	Juice of root is taken in case of fever.	Glucosides, tannins [124]	0.04	0.13
*Foeniculum vulgare* Mill. Apiaceae	ISL 103476	Sonf	Herb	Seeds	Decoction, infusion	**Stomach disorders**, diarrhea	10 g seeds boiled in 100 ml water and decoction prepared which is used to cure stomachache and diarrhea.	Glucoside, saponins, alkaloids [125]	0.09	0.17
*Fragariavesca* L. Rosaceae	ISL-106789	Budhamewa	Herb	Leaves, fruit	Juice	**Menorrhagia**	Juice of leaves is extracted and taken at least once in a day in case of menstrual pain.	Phenolics, Flavonoids [126]	0.05	0.17
*Gentiana kurroo* Royle. Gentianaceae	ISL-67293	Pashanbhed	Herb	Leaves, flower	Decoction	**Blood tonic**	Decoction of leaves and flowers are used as a blood purifier.	Phenolics, flavonoids and proanthocyanidins [127]	0.04	0.2
*Geranium wallichianum* D.Don ex Sweet Geraniaceae	ISL-101166	Ratanjote	Herb	Flower	Tea	**Rheumatism**	Flowers are boiled with water and mixture is stained. This tea is taken in the morning for treating joint pain.	Steroids, coumarins [128]	0.03	0.09
†*Gerbera gossypina* Royle Asteraceae	ISL- 96899	Ladrun	Herb	Root	Juice	Blood pressure, **gastric diseases**	Root juice consumed to lower the blood pressure.	Alkaloids, flavonoids [129]	0.08	0.11
†*Geum adnatum* Wall. Rosaceae	ISL-97938	Shoonkar	Herb	Root	Decoction	**Dysentery**, diarrhea	Decoction of root is consumed orally in case of chronic dysentery or diarrheal bleeding.	Sterols, terpenoids [130]	0.04	0.09
†*Isodon rugosus* (Wall. ex Benth.) Codd Lamiaceae	ISL-16965	Peemar	Shrub	Leaves	Juice	**Diarrhea**	Leaves juice is extracted with water and is given to treat diarrhea two times a day.	Alkaloids, glycosides, flavonoids, terpenoids, anthraquinones [131]	0.04	0.05
*Justicia adhatoda* L. Acanthaceae	ISL 13237	Baikar	Shrub	Leaves	Decoction	**Bronchial diseases**	Decoction of leaves is taken to give ease in cough and bronchial disorders.	Alkaloids, tannins [132]	0.03	0.07
†*Laphangium affine* (D. Don) Tzvelev Asteraceae	ISL-40456	Janglidodal	Herb	Leaves	Decoction	**Influenza**	5 g of leaves are boiled in water. Decoction prepared is cooled and put into bottle. This is used from time to time in case of flu and cold.	Flavonoids, alkaloids, terpenes [133]	0.03	0.17
†*Leucas cephalotes* (Roth) Spreng. Lamiaceae	ISL 16961	Chara	Herb	Leaves	Decoction	**Malaria**	Decoction is prepared and consumed in case of malaria.	Sterols, flavonoids [134]	0.04	0.09
*Malva neglecta* Wall. Malvaceae	ISL 66146	Sonchal	Herb	Leaves	Decoction	**Constipation**	Leaves boiled in water are frequently consumed in case of constipation.	Alkaloid, flavonoid, saponin [135]	0.02	0.12
*Mentha arvensis* L. Lamiaceae	ISL 116029	Podina	Herb	Leaves	Infusion	Diarrhea, **vomiting**	Dried leaves are infused in boiling water and taken in case of diarrhea and vomiting.	Alkaloids, flavonoids, tannins, phenols, cardiac glycosides [136]	0.06	0.07
*Mentha longifolia* (L.) Huds. Lamiaceae	ISL 116056	Babri	Herb	Leaves, Flower	Tea	Abdominal diseases, **vomiting**, nausea	Leaves are dried and then boiled with water to make tea which is used in vomiting.	Terpenoids, flavonoids [137]	0.07	0.21
*Morus alba* L. Moraceae	ISL-10625	Chitta toot	Tree	Bark	Decoction	**Asthma**	Bark is chopped into pieces, boiled in water and decoction is given to get relief in severe cough.	Phenolics, flavonoids [138]	0.03	0.09
*Morus nigra* L. Moraceae	ISL-10697	Kala toot	Tree	Fruit	Decoction	**Throat pain**	Fruit decoction is prepared and gargled in sore throat.	Phenolics, flavonoids [138]	0.05	0.17
†*Muehlenbeckia hastulata* (Sm.) I.M. Johnst. Polygonaceae	ISL-97865	KhattaHulla	Herb	Leaves	Juice	**Bleeding**	10–15 g leaves are put in water for 2 h. Juice obtained is taken in case of internal bleeding.	Anthraquinones [139]	0.03	0.11
*Nerium oleander* L. Apocynaceae	ISL-47361	Gandeera	Herb	Leaves	Decoction	Stomach ailments, **constipation**	Half kg branches boiled in 2 L of water for 2 h in order to make decoction. Two teaspoon of this decoction used daily for constipation and stomach pain.	Tannins, saponins, terpenoids [140]	0.1	0.15
*Ocimum basilicum* L. Lamiaceae	ISL 16251	Neazbu	Herb	Whole plant	Juice	**Stomach diseases**	Juice of plant is consumed in case of stomach disorders.	Phenolics [141]	0.04	0.07
*Oenothera rosea* L Her. ex Aiton Onagraceae	ISL 10057	Jungligulab.	Herb	Leaves, stem	Infusion	**Constipation**	Plant material is infused in boiling water and taken in case of constipation.	Carbohydrates, tannins, saponins, and steroids [142]	0.04	0.15
*Olea europaea* (Wall. and G.Don) Cif. Oleaceae	ISL-116120	Kaahu	Tree	Leaves	Decoction	Toothache, **infections**	Decoction of leaves is prepared and used in gargling in toothache.	Flavonoids, tannins [143]	0.06	0.11
†*Onopordum acanthium* L. Asteraceae	ISL-98635	Kandyara	Herb	Leaves, stem	Decoction,juice	**Ulcers**	Stem decoction is preferably consumed to treat stomach ulcers.	Terpenoids [144]	0.02	0.12
*Onosma bracteata* Wall. Boraginaceae	ISL-95864	Gaozaban	Herb	Leaves	Decoction	**Stomach disorders**	Leaves infusion is consumed after regular intervals until get relief from stomachache.	Tannins, terpenoids, flavonoids [145]	0.04	0.09
*Oxalis corniculata* L. Oxalidaceae	ISL-38367	Khattibooti	Herb	Whole plant	Infusion	**Stomach disorders**	Whole plant powder is mixed in hot water with addition of sugar, stained, and given for stomach ailments.	Carbohydrates, glycosides, phenols [146]	0.02	0.12
*Paeonia emodi* Royle Paeoniaceae	ISL-91236	Mamaikh	Herb	Root	Decoction	**Blood purification**	Roots are boiled in water and taken as a remedy to purify blood.	Flavonoids, sterols, saponins [147]	0.02	0.35
*Periploca aphylla* Decne Apocynaceae	ISL-22591	Batta	Shrub	Bark	Decoction	**Stomach disorders**	Bark decoction is taken to give relief in stomach disorders.	Alkaloids, flovonoids, terpenoids [148]	0.02	0.13
*Persicaria alpine* (All.) H.Gross Polygonaceae	ISL-106743	Masloon	Herb	Seeds	Infusion	**Diarrhea**, fever	Seeds are infused in water. The mixture is stained and kept in a bottle. This mixture is consumed daily in case of fever.	Terpenoids, flavonoids [148]	0.1	0.15
†*Pimpinella diversifolia*DC. Apiaceae	ISL 103476	Tarpakhi	Herb	Leaves	Decoction	Cough, **cold**	Leaves decoction is consumed in cough and cold.	Phenols, glucosides [149]	0.04	0.09
*Punica granatum* L. Punicaceae	ISL 87324	Daroona	Shrub	Fruit	Juice	**Stomach ailments**	Seeds are soaked in water for a day then filtered the mixture. Add sugar and taken twice to treat stomach aches.	Alkaloids, flavonoids, tannins, saponins [150]	0.02	0.18
*Quercus oblongata* D.Don Fagaceae	ISL-10672	Reen	Tree	Bark	Decoction	**Rectal disorders**	Decoction is made by using ground bark powder and given for intestinal disorders.	Tannins, terpenoids, steroids, alkaloids [151]	0.05	0.17
*Rheum australe* D.Don Polygonaceae	ISL-98765	Chutial	Herb	Root	Decoction	**Acidity**	Roots are boiled in water and given orally to treat acidity.	Anthraquinones [152]	0.02	0.09
*Rubus fruticosus* L. Rosaceae	ISL-106878	Aakhray	Herb	Leaves, roots	Infusion, decoction	**Dysentery**, diarrhea, bleedings, whooping cough	Leaves are boiled in water and consumed in case of cough. Decoction of flowers is also consumed in dysentery.	Phenolics, tannins [153]	0.1	0.1
†*Smilax aspera* L. Smilacaceae	ISL-11624	Shogr	Herb	Whole plant	Decoction	**Infection**	Decoction of whole plant is prepared and cooled. It is used to treat infection.	Phenolics, flavonoids [154]	0.02	0.07
*Solanum nigrum* L. Solanaceae	ISL 112661	Kachmach	Herb	Leaves, flower, fruit	Juice	Rheumatism, cough, fever, **bronchitis**, diarrhea	The juice of the leaves can be used alone or with addition of honey to cure diarrhea and cough.	Alkaloids, saponins, tannins [155]	0.1	0.16
† *Solanum surrattense* Burm. f. Solanaceae	ISL-67895	Mokri	Herb	Leaves, fruit	Infusion	**Body pain**	Fruit peels are infused in water and consumed for a week to get relief from body pain.	Terpenes, phenolics, quinones [156]	0.01	0.21
†*Solanum virginianum* L. Solanaceae	ISL-10237	Ghanar	Herb	Leaves	Infusion	**Skin diseases**	Infusion of leaves is taken once in a day for skin diseases.	Alkaloids, flavonoids [156]	0.05	0.09
*Sonchus asper* (L.) Hill Asteraceae	ISL-10987	Dodhal	Herb	Leaves	Infusion	**Abdominal diseases**	Leaves infusion is taken in case of abdominal pain.	Steroids, glycosides, flavonoids [157]	0.02	0.13
*Swertia petiolata* D. Don Gentianaceae	ISL-33193	Chirayetta	Herb	Leaves, stem, root	Decoction	Ulcers, asthma, **urinary disorders**	Decoction of plant is given twice a day in case of urinary disorders.	Terpenoids, flavonoids, alkaloids [158]	0.16	0.12
*Tagetes minuta* L. Asteraceae	ISL-16983	Satbarga, Gainda	Herb	Leaves, flower	Juice	**Earache** and ophthalmic	Leaves and flowers juice is extracted with water and taken orally in case of earache.	Saponin, tannin, alkaloid [159]	0.13	0.13
*Tamarindusindica* L. Fabaceae	ISL-10045	Imli	Tree	Fruit	Infusion	**Fibril diseases**	Peels of fruit are infused in water and taken to treat fibril disorders.	Alkaloids, anthraquinone, glycoside [160]	0.04	0.17
*Taraxacum officinale* F.H.Wigg. Asteraceae	ISL-116231	Peelibooti	Herb	Leaves	Decoction	**Constipation**, jaundice	Leaves are boiled in water and consumed to treat constipation.	Glucosides, terpenoids, tannins [161]	0.04	0.13
*Trichodesma indicum* (L.) Sm. Boraginaceae	ISL-116018	ChotaKulfa	Herb	Leaves	Juice	Eye infections, **dysentery**	Juice of leaves is taken with addition of sugar and salt as per taste to treat dysentery.	Steroids, terpenoids, lipids [162]	0.04	0.08
*Trifolium repens* L. Fabaceae	ISL 123169	Shatal	Herb	Flowers, leaves, root	Infusion	Fevers and **leucorrhoea**	In case of leucchorea the infusion of flowers and leaves is taken.	Flavonoids, steroids [163]	0.08	0.05
*Valeriana jatamansi* Jones ex Roxb. Caprifoliaceae	ISL 18643	Murma	Herb	Root	Decoction	Headache	Decoction of 3-4 g of roots is consumed in case of headache.	Flavones, Glycosides [164]	0.03	0.14
*Verbascum thapsus* L. Scropulariaceae	ISL 11315	Janglitambaku	Herb	Leaves	Infusion	**Skin problems**	Boiled water is poured over the leaves and kept for 2 h then taken orally for skin diseases and skin allergy.	Glycosides, flavonoids, terpenoids [165]	0.02	0.1
*Verbena officinalis* L. Verbenceae	ISL 114005	Neelgu	Herb	Whole plant	Decoction	**Joint pain**	Decoction is prepared and taken once a day for rheumatic disorders.	Flavonoids, Glucosides, Phenolics [166]	0.06	0.14
*Viola canescens*Wall. Violaceae	ISL 40466	Banafsha	Herb	Leaves	Decoction	**Respiratory disorders**	Leaves are boiled in water and taken orally for respiratory disorders.	Alkaloids, sterols, flavonoids [167]	0.04	0.15
†*Vitis vinifera* L. Vitaceae	ISL 59527	Daakh	Climber	Fruit	Juice	**Cough**, asthma	Fresh fruit juice is used directly in case of cough.	Alkaloids, flavonoids, saponins, tannins [168]	0.04	0.14
*Zanthoxylum armatum* DC. Rutaceae	ISL 11640	Timber	Shrub	Seeds, bark	Infusion	**Indigestion**, hemorrhoids, ulcers	Pour a cup of boiling water onto 1–2 teaspoonful of the bark+ fruit and let infuse for 10–15 min and used for stomach pain.	Anthraquinone, glycoside flavonoids [169]	0.04	0.15
*Ziziphus nummularia* (Burm.f.) Wight and Arn. Rhamnaceae	ISL 11625	Beri, Jand	Shrub	Root	Infusion	**Diabetes**	Boil 5–8 g of grinded root with 1 glass of water till volume of water reduced to half. It is used to cure diabetes.	Alkaloids, flavonoids, phenolics [170]	0.1	0.1

In column scientific name, species name starting with † indicates newly reported species. *NH* National Herbarium, *UV* use value, *RFC* relative frequency of citation.

^††^Uses in bold indicate preferred uses for a given plant

#### UV of HD plant species

The UV of species varied from 0.0039 to 0.2196. The first 14 species with the highest values of UV are summarized in Table 3, with *V. jatamansi*, *I. rugosus*, *O. acanthium*, *A. nilotica*, and *V. canescens* being the top 5 most used species. The results of the models run to test how their UV varied across gender and age categories are summarized in Table 3 too. For all the species (except *V. jatamansi*), UV differed insignificantly (*p* value > 0.05) between men and women and between age categories. UV of *V. jatamansi* was lower for men (est = − 0.88, se = 0.38, prob. = 0.021).

**Table 3:** Relationship between uses of the 14 species with the highest UV and gender and age of informants: summary of the Poisson GLM (SE = standard error, − absent from the parsimonious model)

Species	UV	Gender^†^: male		
		Estimate	SE	*p* value
*Valeriana jatamansi* Jones ex Roxb.	0.2196	− 0.881	0.381	0.021
*Isodon rugosus* (Wall. ex Benth.) Codd	0.0980	–	–	–
*Onopordum acanthium* L.	0.0392	–	–	–
*Acacia nilotica* (L.) Willd. ex Delile	0.0275	–	–	–
*Viola canescens* Wall.	0.0275	–	–	–
*Rubus fruticosus* auct. (L.)	0.0235	–	–	–
*Leucas cephalotes* (Roth) Spreng.	0.0235	–	–	–
*Mentha arvensis* L.	0.0235	–	–	–
*Achillea millefolium* L.	0.0196	–	–	–
*Persicaria alpina* (All.) H. Gross	0.0196	–	–	–
*Fagopyrum esculentum* Moench	0.0196	–	–	–
*Solanum nigrum* L.	0.0196	–	–	–
*Ziziphus nummularia* (Burm.f.) Wight and Arn	0.0196	–	–	–
*Morus alba* L.	0.0196	–	–	–

^†^Female taken as reference

### Medicinal uses of HDs in Azad Jammu and Kashmir

One hundred and eleven (111) diseases grouped into 13 ailment categories were reported to be cured using HDs (Table 4). The most frequently cited diseases were gastrointestinal (GIT) diseases, respiratory diseases, and fevers. The least cited diseases were urinary disorders, oral diseases, and infectious diseases (Table 4).

**Table 4:** Disease categories treated by herbal drinks

Diseases	Local names of diseases	Relative frequency of citations (%)
Gastrointestinal (GIT) diseases	Aantoonkebemaryaan	17.43
Respiratory diseases	Tanafsibemaryaaan	12.60
Fevers	Bukhaar	9.92
Nail, skin, and hair disorders	Jildi bemaryaan	8.58
Ear, nose, eye diseases	Kann, naak,aankhkebemaryaan	8.04
Glandular disorders	Ghadoodibemaryaan	7.51
Nervous disorders	Aasabibemaryaan	6.97
Sexual diseases	Jinsibemaryaan	6.97
Cardiovascular diseases	Qalbibemaryaan	6.43
Muscle and skeletal disorders	Pathoonkebemaryaan	6.17
Urinary disorders	Faazlatibemaryaan	3.49
Oral diseases	Moonhkebemaryaan	3.22
Infectious diseases	Wabaiamraaz	2.68

**Fig. 5:**
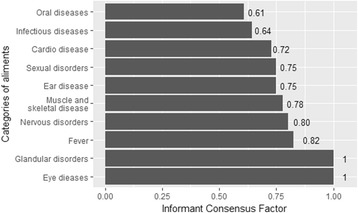
Informant consensus factor for diseases treated by herbal drinks in Azad Jammu and Kashmir

For all 10 ailment categories, values of IFC were all greater than 0.60 (Fig. 5) indicating that relatively few species were used by a large proportion of informants for each category of ailment. In paricular, only one species, *Quercus oblonga,* was used for “glandular disorders,” and only one other, *Tagetes minuta*, was used for “eye diseases” (Fig. 5).

## Discussion

This study conducted within the indigenous communities of the region of Azad Jammu and Kashmir, Pakistan (i) assessed the diversity and life forms of plants used in HDs and tested for generation and gender differences in terms of known HD plant species; (ii) identified the most commonly used plants for HDs and the species-specific uses relationship with gender and generation; (iii) documented plant parts used in HD preparations, preparation mode, and mode of utilization; and (iv) determined the diseases treated by HDs and the diversity of plants used for each disease.

### HD plant diversity and life forms

Most of the people inherited traditional knowledge from their elders and continued to transmit it from one generation to another [48]. Female informants were less numerous in the present study, owing to the reason that they were reluctant to give information, and used plants in homes and at a domestic level only. Among the reported plant families, Asteraceae was the most used in the treatment of various diseases. The reason for dominance of this family in plants used for HDs may be that the plants belonging to this family are naturally diverse and widespread in the study area [49, 50]. Asteraceae is also the largest plant family in the world including over 1600 genera and 23,000 individual species, many of which are important for medicinal uses. This pattern is consistent with the non-random plant selection theory, which predicts that the number of medicinal species used in a botanical family in a given region would be linearly related to the total number of plant species in that family [51, 52]. Therefore, these plants are easily available and are consumed locally for various purposes, particularly medicinal uses. Family Asteraceae was also reported elsewhere to be the largest plant family in terms of the number of medicinal plants used in the treatment of various ailments [53, 54]. Such a similarity may be due to the presence of biologically active chemical constituents that make them medicinally important [55].

Herbs were exploited preferably than shrubs and trees because of their availability in addition to their high effectiveness for treatment of diseases than other growth forms. The frequent utilization of herbaceous medicinal plants was also described in studies conducted in various regions of the world [27, 56–59]. The reason for extensive utilization of herbs may be due to their easy accessibility as well as their therapeutic properties [60–62], as supported by the availability hypothesis, which predicts that plants are used for medicine because they are more accessible or locally abundant [63, 64].

### Age- and gender-related knowledge of plants used for HDs

Several informants’ socio-cultural and demographic characteristics, in particular gender and age, are correlated with plant knowledge [32, 33]. Such a relationship formally known as the age, gender, and dynamics of knowledge hypothesis [28] is specific to the type of knowledge studied but is also context-specific [28]. Whereas some authors found that men are more knowledgeable than women [45, 65], others found the contrary, especially where women served as the primary healthcare providers in their families [34, 66]. Similarly, older people tend to have greater knowledge of the local medicinal flora [34, 67] because the accumulation of medicinal plant knowledge is a life-long process [32]. Here, we found no evidence for either quantitative or qualitative differences between men and women or between the two generations considered (< 40 years, ≥ 40 years). The lack of difference between men and women was also reported in the Sahelian zone of Africa [68] and may reflect the fact that both men and women are equally involved in health-related issues not only at a household level but also at a community level. However, the lack of difference among generations may be because HDs are common practices in the region and are accessible to everyone including youngers or because our sample did not include enough younger informants to capture such a trend. (All informants were aged at least 22 years, and only 17 informants were between 22 and 29 years).

### Treated disorders, plant part used, and forms of uses

The people of Azad Jammu and Kashmir used HDs against 13 diseases. The finding that GIT diseases, respiratory diseases, and fevers were the most cited may suggest that these disorders are the most frequent in the study area. Different plant parts are used in treating various ailments depending on the type and condition of ailment treated. These plant parts are reported to accumulate a variety of phytochemicals that are important for treatment of various disorders by local communities of people [69, 70]. Leaves were found to be the most used plant part likely because leaves are easy to collect as compared to other plant parts [71] but also because leaves possess chemical constituents in larger quantities, which may be responsible for medicinal properties [72]. This information is in accordance with studies conducted in different regions of Pakistan [73, 74]. After leaves, roots, which are also known to possess high concentrations of chemicals [75, 76], were the second most used plant part in this study. The main form of preparation reported by informants was decoction. Decoctions are easier to prepare because plant parts are only to be boiled with water [73]. Mostly, fresh plant parts are preferably used in preparation of HDs. Informants reported that drying the plant parts may cause their efficacy to decrease because the ingredients present are damaged to some extent. Both modes of preparation and prescriptions are commonly used in traditional herbal medicine, and similar results have been presented in previous ethnobotanical records that were carried out in the study area [77–80].

The medicinal HDs reported in this study for treatment of different diseases can also be exploited further for their pharmacological and phytochemical properties. Previous ethno-botanical studies conducted in various parts of the study area also reported recurrent practice of using medicinal plants against GIT ailments [38, 81]. Other conspicuous disorders cured by medicinal HDs include cardiovascular diseases, ear, nose, and eye diseases, glandular disorders, infectious diseases, muscle and skeletal disorders, nervous disorders, oral diseases, sexual diseases, and urinary disorders. The lowest cited ailments in this study were urinary disorders suggesting a weak prevalence of urinary problems in the study area. One of supporting reasons may be that people living in villages mostly use spring water or water from wells, which is clean from many impurities, so they have less chance of developing kidney or urinary problems. A number of disorders have been reported from the study area that are treated using medicinal HDs, and this could be attributed to lack of health facilities in remote and high-altitude areas.

In the present study, medicinal plants used as HDs were reviewed for the presence of different phytochemicals reported in previous literature (see Table 2, column “Active constituents”). Almost all the plants had been reported earlier for their one or more phytochemical compounds indicating their significance in medicinal treatments. The highest number of phytochemicals was reported for *Bauhinia variegata*, *Bergenia ciliata*, *Isodon rugosus*, *Mentha arvensis*, *Oenothera rosea*, *Punica granatum*, and *Vitis vinifera*. This information would be helpful in authenticating the use of these species as herbal drugs in curing various ailments and could be explored for their detailed phytochemical, biological, and pharmacological aspects to facilitate the scientific community in new drug discovery [82]. Flavonoids and alkaloids were reported in most of the HD species, indicating their significance. Flavonoids have been reported for their significant effects against many ailments and disorders such as inflammation, allergy, and thrombic disorders [83]. The presence of alkaloids could be related to the indication of cytotoxic, antibacterial, and analgesic properties in plants [84]. Pharmaceutical ethnobotanical studies are still very useful for identifying novel or scarcely reported medicinal uses of plants, which could provide the foundation for new drugs synthesis [85].

The values of ICF per disorder suggested that relatively few species were used for each ailment category, with only one species, namely, *Quercus oblonga*, used for glandular disorders (disorders cited by 7.51% of informants) and only one other, namely, *Tagetes minuta*, for eye diseases (disorders cited by 8.58% of informants). Infectious diseases (ICF = 0.64) and oral diseases (ICF = 0.61) had the lowest ICF indicating a more diverse use of plant species compared to other disease categories. Based on the utilitarian redundancy model, species sharing the same therapeutic function are redundant and are predicted to experience a reduced use-impact because the use pressure is diffused across a greater number of species [86]. Thus, all things being equal, the loss or rarefaction of one species in ailment categories with lower ICF is predicted to have little overall effect on the ethnomedicinal practices in the region [87]. On the contrary, particular conservation attention should be paid to the single species used for eye diseases and glandular disorders.

### Most used species: medicinal uses and gender and generation effects

The top five most used species for HDs were *V. jatamansi*, *I. rugosus*, *O. acanthium*, *A. nilotica*, and *V. canescens*. Their high UV may be due to their easy accessibility, availability, wide distribution in the area, and the strong ethnic culture to use these plants for medicinal purposes [88]. The medicinal and socio-economic importance of these species has been highlighted by many previous studies including ethno-pharmacology prospections in the Himalayan region. *V. jatamansi* is a well-known medicinal species in Asia [89, 90]. The species is used in several Ayurvedic preparations and is known to cure obesity, skin diseases, insanity, epilepsy, and snake poisoning [91]. The essential oil and extract of the species is used in the flavoring, pharmaceutical, and fragrance industries, and about 30 products are commercially available [91]. *I. rugosus* is extensively used as TM for the management of various types of pain including tooth ache, gastric pain, abdominal pain, ear ache, and generalized body pain [92, 93]. *O. acanthium* has application in medical practice as a bactericide, cardiotonic, and hemostatic agent, is used against hypotonicity [94, 95], and is a great bio-oil source [96]. *O. acanthium* is sometimes sold as an ornamental plant and has reportedly been used to treat cancers and ulcers and to diminish discharges of mucous membranes [96].

*A. nilotica* is a widespread multipurpose tree used extensively for the treatment of various diseases, e.g., colds, bronchitis, diarrhea, dysentery, biliousness, bleeding piles, and leukoderma [97], and its bioactivity has been proven by many studies [94, 98]. *V. canescens* is commonly used as TM in the north-west Himalaya for the treatment of protozoan infections and fever including malaria [21], and its anti-malarial activity was proven by Verma, Dua [99].

A significant relationship of the UV with gender was found for only 1 species out of the top 14 most used species, namely, for *V. jatamansi*. This finding corroborates the hypothesis that the effect of gender and age category on the medicinal uses of plant species is species-specific and context-specific [28].

### Novelty and added value for the study region

The comparison of species reported in the present study with those in previous literature of the same region revealed 17, i.e., 22%, new species used as HD species including *Adiantum venustum* D. Don, *Euphorbia illirica* Lam., *Fagonia cretica* L., *Fagopyrum esculentum* Moench, *Gerbera gossypina* Royle, *Geum adnatum* Wall., *Isodon rugosus* (Wall. ex Benth.) Codd, *Laphangium affine* (D.Don) Tzvelev, *Leucas cephalotes* (Roth) Spreng, *Muehlenbeckia hastulata* (Sm.) I.M. Johnst., *Onopordum acanthium* L., *Pimpinella diversifolia* DC., *Smilax aspera* L., *Solanum surattense* Burm. f., *Solanum virginianum* L., and *Vitis vinifera* L. This could be linked to the fact that *TM* is a dynamic system and is based on a recurrent trial and error process that is transmitted across generations and guides further use of plants [100, 101].

### Implications for public health and environmental policies

From the results provided, GIT disorders (stomach related health problems) and respiratory disorders (cough, bronchitis) were the most prevalent health problems in the study area. Stomach disorders probably spread in these areas due to malnutrition and unhygienic use of food stuffs. Respiratory problems can be attributed to the high altitude of the study area, where the air is cold. People traditionally use food medicines to treat such diseases, which in many cases are quite effective. Therefore, the present findings suggest that public-health administrators should devise some health policies regarding the general health problems in the study area and the TM practiced by the indigenous community of the area as part of their primary healthcare.

## Conclusion

Recently, the utilization of medicinal plants has gained much attention due to their fewer side effects as herbal remedies. The study area has a diversity of medicinal plants, which are still used against many ailments, but there is lack of traditional knowledge transfer among the new generation. From ancient times, the use of hot watery infusions made from wild or cultivated plants have been employed as a remedy for a number of ailments. In present study, 72 medicinal plants used as HDs (decoction, infusion, tea, juices) for curing various ailments were documented. The most preferred form of utilization was decoction, and herbs were the dominant life form exploited. Leaves were used mostly in preparation of medicinal HDs. The key informants were mostly male between 40 and 50 years old, and some herbalists also provided the required information. The wealth of original knowledge obtained from the present work strengthens the significance of expanding the study to other parts of the study area. The therapeutic uses of the HDs provided basic data for further research focused on phytochemical and pharmacological studies and conservation of the most important species.

## Abbreviations

ANOSIM: Analysis of similarities
GLM: General linear model
HD: Herbal drink
ICF: Informant consensus factor
RFC: Relative frequency of citation
TM: Traditional medicine
UV: Use value
